# Histamine H3 Receptor Antagonists Influence the Directional Growth of Type II Spiral Ganglion Neurites Within the Developing Cochlea of C57BL/6 Mice

**DOI:** 10.1007/s11064-025-04521-9

**Published:** 2025-08-13

**Authors:** Lingyi Kong, Heidi Olze, Agnieszka J. Szczepek

**Affiliations:** 1 Department of Otorhinolaryngology, Head and Neck Surgery, Charité- Universitätsmedizin Berlin, Corporate Member of Freie Universität Berlin and Humboldt-Universität zu Berlin, 10117 Berlin, Germany; 2 Faculty of Medicine and Health Sciences, University of Zielona Góra, Zielona Góra, 65-046 Poland

**Keywords:** Histamine H3 receptor, Cochlea, Hair cells, Spiral ganglion neurons, Antagonists

## Abstract

The histamine H3 receptor (H3R) is a crucial regulator of synaptic plasticity, neurotransmitter release, and neural signaling within the central nervous system. However, its role in the cochlea remains poorly understood, even though mast cells, a rich endogenous source of histamine, have recently been documented in the mammalian cochlea. This study examined H3R expression and localization in the postnatal day 4–5 (P4-5) C57BL/6 mouse cochlea and evaluated its functional consequences under antagonist treatment. RT-qPCR analysis showed significantly higher *H3R* mRNA levels in the modiolus compared to the organ of Corti and the lateral wall. Immunofluorescence staining confirmed H3R localization in hair cells (HCs) and spiral ganglion neurons (SGNs). Dissected cochlear explants exposed to two distinct H3R antagonists—ciproxifan and pitolisant—at concentrations of 10µM, 50µM, and 100µM, displayed different responses: ciproxifan induced dose-dependent HC loss. In contrast, pitolisant caused no loss of HC but led to stereociliary abnormalities at higher concentrations. Both antagonists disrupted type II SGN neurite projections, redirecting their normal basal-directed trajectory toward the apical region. These findings implicate H3R in maintaining cochlear structural integrity and guiding SGN neurite development during early postnatal maturation. Further investigation into H3R-mediated mechanisms may reveal new therapeutic targets for hearing preservation and repair.

## Introduction

The cochlea is a highly specialized and tonotopically organized structure in the mammalian inner ear that converts acoustic stimuli into neural signals [1, 2]. This transformation is mediated by the coordinated function of mechanosensory hair cells (HCs) in the organ of Corti and spiral ganglion neurons (SGNs) [1, 3]. HCs transduce sound-induced mechanical vibrations into electrical signals, which SGNs transmit to the central auditory pathways [1, 4]. At the apical surface of each HC lies a bundle of actin-rich stereocilia, arranged in a staircase-like pattern and interconnected by tip links [4, 5]. Deflection of these bundles opens mechanotransduction (MET) channels, initiating the receptor potential and neurotransmitter release [6, 7]. The cochlea’s unique structural composition—comprising fluid-filled compartments, specialized epithelial domains, and an electrochemical gradient maintained by the stria vascularis—provides the biophysical environment necessary for this sensory conversion [8, 9, 10, 11]. During early postnatal development, the cochlea undergoes extensive morphological and functional maturation, including refinement of HC-SGN synapses, stereocilia alignment, and regional specialization along the cochlear axis [12, 13, 14, 15]. Disruption of these processes can compromise auditory encoding and lead to permanent hearing impairment.

Histamine is a bioactive amine involved in various physiological processes, including immune responses, inflammation, and neurotransmission [16, 17, 18]. It is synthesized from histidine via histidine decarboxylase (HDC) and exerts its effects by binding to four G protein-coupled receptors: histamine H1 receptor (H1R), histamine H2 receptor (H2R), histamine H3 receptor (H3R), and histamine H4 receptor (H4R) [17, 19, 20]. Among them, the H3R is predominantly expressed in the central nervous system and serves as both an autoreceptor and heteroreceptor [21, 22]. Through coupling to Gi/o proteins, H3R reduces intracellular cAMP levels and inhibits calcium influx, thereby modulating the release of histamine and other neurotransmitters such as dopamine, acetylcholine, and GABA [23, 24]. It is critical in regulating synaptic plasticity, sleep–wake cycles, memory, and attention [23, 25, 26].

Pharmacologically, H3R can be influenced by full agonists, neutral antagonists, or inverse agonists (in most clinically used compounds) that stabilize the receptor in its inactive state [27]. When H3R is blocked, its autoinhibitory control on presynaptic terminals is lifted, leading to greater histamine and co-transmitter release and enhancing cortical arousal [28]. Pitolisant, the first H3R antagonist approved for clinical use, is licensed for the treatment of narcolepsy and promotes wakefulness by enhancing histaminergic neuronal activity [29]. Beyond their excitatory effects, antagonism of H3R also attenuates neuroinflammation by limiting microglial and astrocytic activation [30]. Conversely, experimental H3R agonism leads to inhibition of the release of neurotransmitters [23]. These dual neuro-immune properties make H3R ligands attractive for neurological disease and chronic inflammatory conditions, highlighting the receptor as a bridge between neural signaling and innate immunity.

Emerging evidence highlights histamine signaling as a potentially critical regulator of inner ear physiology. In 2020, our research group first identified mast cells—a principal source of histamine—in the cochleae of rats and mice, suggesting that histamine may play previously unrecognized roles in cochlear function and immune regulation [31]. Upon activation (e.g., via IgE-mediated stimulation), mast cells release a wide array of mediators, including histamine, which has been shown to modulate inflammation, vascular permeability, and neuronal excitability [32, 33, 34]. Wu et al. demonstrated that mice lacking histidine decarboxylase (HDC) exhibited significantly increased susceptibility to cisplatin-induced hearing loss, which could be reversed by exogenous histamine administration [35]. These findings underscore a potentially protective role of histamine in the cochlea, highlighting the need to explore the molecular pathways through which histamine and its receptors contribute to cochlear homeostasis and resilience to injury.

There are two potential sources of histamine in the cochlea: resident mast cells [31] and the auditory hair cells, which express histidine decarboxylase – the only enzyme responsible for producing histamine [35]; however, the concentration of histamine in the inner ear was never determined. In the in vitro experimental settings, histamine is absent from the cochlear tissue culture medium. After 24 h in culture, an average of 22.5 nM of histamine can be detected in the tissue culture dish containing one explant and 500 µL of medium (unpublished data, manuscript in preparation), indicating the release of histamine from the cochlear tissues.

Previous work by Takumida et al. described H3R-like immunoreactivity in the adult CBA/J mouse cochlea [36]. Building on that foundation, we mapped the spatial distribution of H3R in the cochlea from C57BL/6 pups using high-resolution confocal microscopy, which offers greater sensitivity, optical sectioning, and precise regional analysis. We also evaluated the effects of two distinct H3R antagonists, ciproxifan and pitolisant, on HC morphology and SGN outgrowth in cochlear explants. The results presented here provide novel insights into the potential role of H3R signaling in early postnatal cochlear maturation, the integrity of stereocilia, and neuronal pathfinding.

## Materials and Methods

### Ethical Approval and Animals

This study was performed in accordance with the EU Directive 2010/63/EU on the protection of animals used for scientific purposes and approved by the Governmental Ethics Commission for Animal Welfare in Berlin, Germany (approval numbers T-CH0036/22 and T-CH0023/24). C57BL/6 pups (P4-5) were obtained from the animal facility at Charité-Universitätsmedizin Berlin and housed under standard conditions. All experimental procedures strictly adhered to the principles of Replacement, Reduction, and Refinement (3R) [37].

### Reverse Transcription Quantitative PCR (RT-qPCR)

C57BL/6 mice (P4-5) were sacrificed by decapitation. After disinfecting the heads with 70% ethanol, they were bisected along the median sagittal plane. Following the removal of brain tissues, the inner ear labyrinth was gently dissected in pre-chilled DMEM/F12 (cat. #21041025, Thermo Fisher Scientific, USA). The tympanic membrane, surrounding cartilage, and cochlear capsule were removed to expose the cochlea. Subsequently, the stria vascularis and spiral ligament were carefully separated, unveiling the basilar membrane upon which the organ of Corti is situated. The basilar membrane was then separated from the modiolus while preserving as much medial tissue as possible to retain the SGNs. Tissue samples were immediately placed in RLT lysis buffer supplemented with β-mercaptoethanol (cat. #4227.3, Carl Roth, Karlsruhe, Germany) and stored at -80 °C until RNA extraction.

Total RNA was isolated using a RNeasy Mini kit (cat. #74106, Qiagen, Germany) following the manufacturer’s protocol. RNA concentration was measured using a NanoDrop^®^ spectrophotometer (OneC, Thermo Fisher Scientific, USA), and purity was assessed by calculating the A260/A280 absorbance ratio, with values between 1.8 and 2.2 indicative of high-quality RNA. QuantiTect Primer Assays (Qiagen, Germany) targeting *H3R* (Mm_Hrh3_1_SG, GeneGlobe ID QT00158375, Transcript NM_133849, mRNA Length 2641 bp) and β-actin (Mm_Actb_1_SG, GeneGlobe ID QT00095242, Transcript NM_007393, mRNA Length 1889 bp) were employed for RT-qPCR analysis. Reactions were performed per the kit guidelines using a LightCycler^®^ 96 system (LightCycler 96 SW 1.1, Roche, Switzerland). Each sample was analyzed in duplicate, and relative gene expression was calculated using the ΔΔCt method.

### Cryosections

For the cryosections, heads of P4-5 C57BL/6 mice were bisected along the median sagittal plane, carefully preserving inner ear structures. The half-head samples were fixed in 4% paraformaldehyde (cat. #TCL119-100ML, Hi-Media, India) at 4 °C for 2 h and rinsed 3 times (5 min each) in 1xphosphate-buffered saline (PBS; cat. #18912-014, Thermo Fisher Scientific, USA) at room temperature (RT). Specimens were subsequently immersed in 30% sucrose solution (cat. #S0389, Sigma-Aldrich, Germany) overnight at 4 °C until fully dehydrated (indicated by sinking). Each specimen was embedded in tissue-freezing medium (cat. #MCKB9180, Sigma-Aldrich, Germany) within cryomolds, carefully avoiding air bubbles, and rapidly frozen at -80 °C.

On the day of sectioning, embedded tissues were equilibrated in a cryostat (CM3050 S, Leica, Germany) for 30 min to prevent brittleness. Sections of 16 μm thickness were cut once the cochlear region was visualized, and structural integrity was confirmed under a light microscope. Prepared slides were stored at -20 °C until subsequent staining.

### Cochlear Explant Culture

#### Preparation and Culture of Cochlear Explants

The cochlear basilar membrane, including modiolus, was dissected as described in Sect. Reverse Transcription Quantitative PCR (RT-qPCR). Immediately after dissection, explants were transferred into 4-well Millicell EZ SLIDES (cat.# PEZGS0416, Merck KGaA, Germany) containing cochlear tissue culture media. The media consisted of DMEM/F-12 (cat.# M23350, Bio-Techne GmbH, Germany) supplemented with 10% fetal bovine serum (FBS; cat. #F4135, Merck KGaA), 0.9% glucose (cat. #G8769, Merck KGaA), 100U/mL penicillin G (cat. #455690050, Thermo Fisher Scientific, USA), 0.2% insulin–transferrin–selenite (cat. #11074547001, Roche, Switzerland), and 24ng/mL mouse IGF-1 (cat. #791-MG, Bio-Techne GmbH). Fresh medium was prepared daily. Experimental groups were treated with the H3R antagonist at various concentrations, while control groups were cultured in plain culture medium. All explants, regardless of group, were maintained under identical conditions at 37 °C in 5% CO₂ for 24 h. Following culture, explants were fixed in 10% formalin solution (cat. #HT5011-15ML, Merck KGaA) for 30 min at RT and stored at 4 °C until immunofluorescence staining.

#### Drug Treatments

Ciproxifan maleate (cat. #184025-19-2, Santa Cruz, USA) was dissolved in dimethyl sulfoxide (DMSO; cat. #20385.01, Serva, Germany) to prepare a 200 mM stock solution, and pitolisant hydrochloride (cat. #HY-12199B, Hycultec, Germany) was dissolved in DMSO to prepare a 100 mM stock solution. All stock solutions were stored at -20 °C until they were used. Cochlear explants were treated with H3R antagonists at final concentrations of 10µM, 50µM, and 100µM.

Control conditions included:

- Vehicle control: Culture medium containing 0.50% or 1.00% DMSO without H3R antagonists.
- Negative control: Culture medium withoutDMSO.

This experimental design enabled a systematic assessment of the effects specifically caused by the H3R antagonists, while accounting for potential solvent-related toxicity.

### Immunofluorescence Staining

#### Cultured Cochlear Explants

Cochlear explants were permeabilized in PBS containing 0.5% Triton X-100 (cat. #39487, Cell Signaling, USA) for 30 min at RT. To reduce nonspecific binding, explants were blocked with 4% normal goat serum (NGS; cat. #005000121, Jackson ImmunoResearch, USA) in PBS for 1 h at RT. The primary antibody (Neurofilament monoclonal Antibody, cat. #orb18247, Biorbyt, UK) was diluted in antibody dilution buffer (340 mM NaCl, 0.1% Triton X-100, 1% NGS, pH adjusted to 7.0) and incubated overnight at 4 °C. The following day, specimens were washed and incubated with appropriate secondary antibody (Goat anti-Mouse IgG Alexa Fluor 488, cat. #A11001, Thermo Fisher Scientific, USA) for 2 h at RT in the dark. Filamentous actin was stained using Phalloidin-iFluor 564 reagent (cat. #ab176753, Abcam, UK) to visualize F-actin-rich hair cells and their stereocilia. Nuclear staining was performed using ProLong Gold Antifade Mountant with DAPI (cat. #8961S, Cell Signaling Technology, USA).

#### Cryosections

Cryosections were air-dried at RT for 20 min and rehydrated in PBS for 10 min. Sections were permeabilized with 0.1% Triton X-100 in PBS for 20 min. Non-specific binding sites were blocked using PBS containing 10% NGS and 0.05% Triton X-100 for 1 h at RT in a humidity chamber. Subsequently, the primary antibodies (Histamine Receptor 3 rabbit polyclonal antibody, cat. #E-AB-13309, Elabscience, China; Neurofilament 200 kDa, cat. #MAB5256A5, Merck, Germany; Ribeye antibody, cat. #192104, Synaptic Systems, Germany) were applied overnight at 4 °C. The manufacturer internally validated the antibody against H3R (rabbit polyclonal, affinity-purified IgG). The antibody against Ribeye (guinea pig polyclonal antiserum) was not validated; however, its use has been documented in seven publications. The mouse monoclonal IgG_1_ against neurofilament 200, directly conjugated with ALEXA555 and internally validated by the manufacturer by immunocytochemistry, has also been used in six publications. Following incubation, sections were washed 3 times in PBS (15 min each) at RT and incubated with secondary antibodies (Goat anti-Rabbit IgG Alexa Fluor 488; cat. #A11034, Thermo Fisher Scientific, USA, Goat anti-Rabbit IgG Alexa Fluor 594; cat. #A11012, Thermo Fisher Scientific, USA; or Goat anti- Guinea pig Alexa Fluor 647; cat. #A-21450, Thermo Fisher Scientific, USA) for 1.5 h at RT in the dark. Filamentous actin marking HC stereocilia was visualized using Flash Phalloidin™ Green 488 reagent (cat. #424201, Biolegend, USA). Nuclei were counterstained with DAPI using ProLong Gold Antifade Mountant with DAPI (cat. #8961S, Cell Signaling Technology, USA). Slides were then mounted using coverslips (24 × 50 mm, Epredia, USA) and stored in the dark until microscopic evaluation.

### Confocal Microscopy and Hair Cell Counting

Images were acquired using a confocal laser scanning microscope (SPE, Leica, Germany) with dry (20x) and immersion oil (40x, 63x) objectives. The lasers at 405 nm, 488 nm, and 552 nm were used for excitation. Image acquisition parameters were set to a pinhole diameter of 1 Airy unit, a frame size of 1024 × 1024 pixels, and a digital scan zoom of 1.5 or 3.5. Z-series acquisition started at the level of the stereocilia tips, identified by peak phalloidin staining at the apical surface of the HCs. It extended basally until the point at which their associated phalloidin fluorescence had completely disappeared. A typical Z-stack was 20 μm thick. Images were processed using LSM Image Browser, LAS AF Lite software, and Adobe Photoshop 2020 (Adobe Inc., USA).

For HC counting, each cochlea was optically divided into ten segments along the apical-basal axis, and microscopic images were captured for each segment, intentionally avoiding regions with handling-related damage. HCs were counted within standardized 150 μm segments. Cells were counted as present if a normal array of stereocilia and intact nuclei were identified; otherwise, they were classified as absent. Data were obtained from at least eight independent cochlear specimens per experimental group, with the highest and lowest counts excluded from analysis to minimize variability.

### Statistical Analysis

Statistical analyses were performed using GraphPad Prism (version 8.0c, GraphPad Software Inc., USA). The Student's t-test was applied when comparing two groups when the data met the assumptions of equal variances. In cases where the data exhibited unequal variances, Welch’s t-test was used. For multiple-group comparisons, one-way ANOVA was conducted, followed by Bonferroni’s multiple comparisons test to correct for multiple testing and control the overall Type I error rate. Data are presented as mean ± SEM, with each *n* representing the number of biologically independent samples. Statistical significance was defined as a p-value less than 0.05, with significance levels *p < 0.05, **p < 0.01, ***p < 0.001, and ****p < 0.0001.

## Results

### Expression of Histamine H3 Receptor in the Neonatal C57BL/6 Mouse Cochlea

**Fig. 1:**
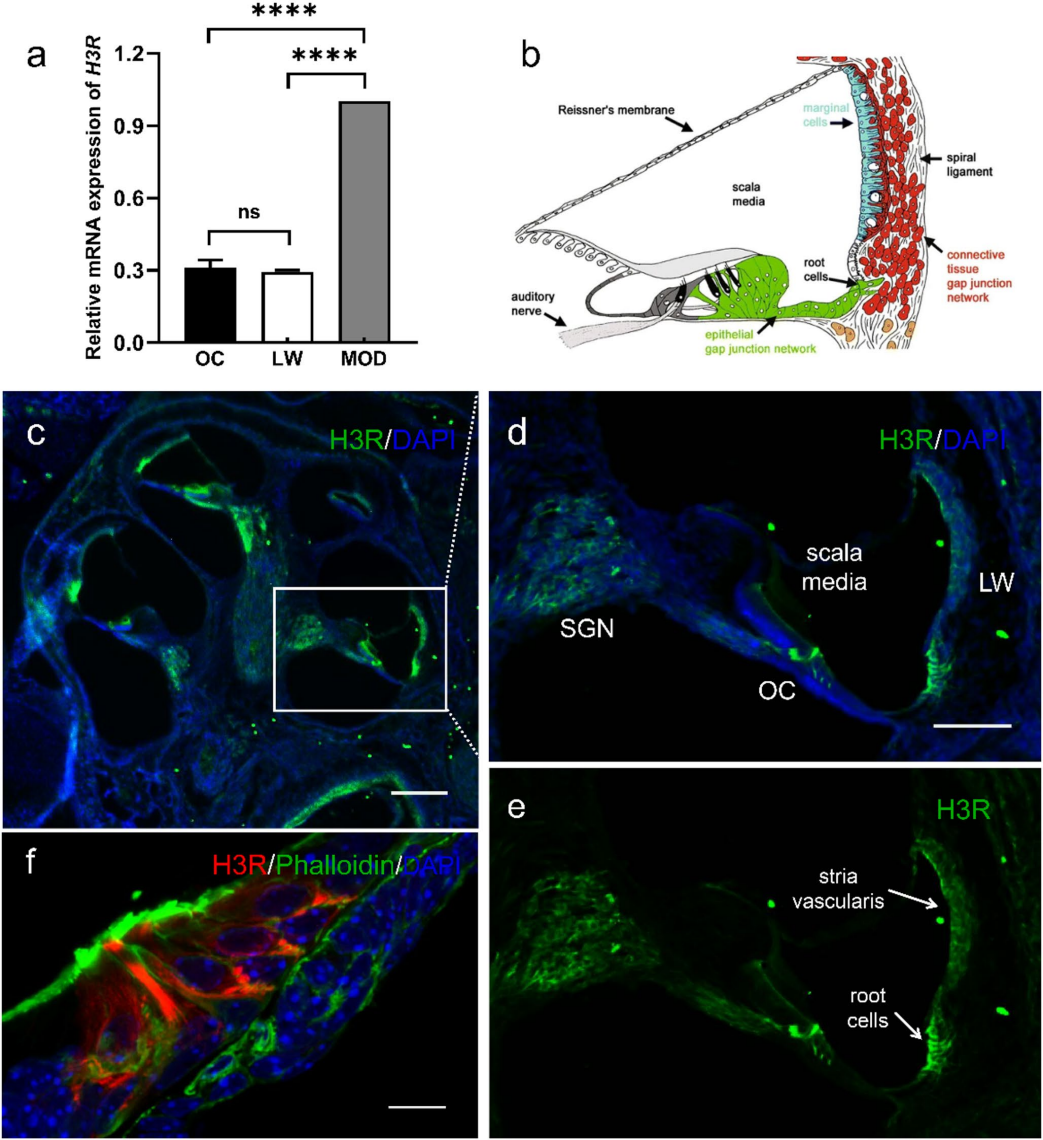
Expression and immunolocalization of histamine H3 receptor (H3R) in the cochlea of P4-5 C57BL/6 mice. **a** Relative *H3R* mRNA expression in the organ of Corti (OC), lateral wall (LW), and modiolus (MOD). Each sample consisted of tissue from four mice, with three biological replicates per group. **b** Schematic illustration of a mid-modiolar section of the mouse cochlea highlighting major anatomical structures within the scala media. Reproduced with permission obtained from Springer Nature under CC BY 4.0 [38]. **c** Low-magnification image of a longitudinal cochlear cryosection. **d**–**e** High-magnification views of the boxed regions in c. **f** Immunofluorescence staining showing co-localization of H3R and phalloidin in both inner and outer HCs within the organ of Corti. H3R (green in c-e, red in f), Phalloidin (green in f), nuclei (DAPI, blue). Scale bars: 200 μm in **c**, 100 μm in **d-e**, 10 μm in **f**

To investigate the distribution of H3R in the neonatal mouse cochlea, RT-qPCR analysis was first performed on three anatomically distinct regions containing the organ of Corti, lateral wall, and modiolus. The region containing the organ of Corti included not only sensory HCs, supporting cells, and pillar cells, but also the basilar membrane and the spiral limbus. The lateral wall comprises the stria vascularis and spiral ligament, and the modiolus primarily contains SGNs and their associated neurites. Our analysis indicated a lack of statistically significant difference in *H3R* mRNA expression between the organ of Corti (0.31 ± 0.03-fold) and the lateral wall (0.29 ± 0.01-fold; ns, *p* > 0.05). In contrast, the H3R expression was markedly higher in the modiolus than in the organ of Corti or the lateral wall (****p < 0.0001), indicating a region-specific enrichment of H3R within the modiolus (Fig. 1a).

To characterize the spatial distribution of H3R protein, cochlear cryosections from P4-5 C57BL/6 mice were stained with the antibody against H3R. H3R was observed in the organ of Corti, the modiolus, and the lateral wall (Fig. 1c-e). In the latter, the root cells and stria vascularis were positive for H3R (Fig. 1e). Within the organ of Corti, immunolabeling was observed in both inner and outer HCs (Fig. 1f). Additionally, we observed a positive signal for H3R at the location corresponding to pillar cells, but the staining varied between specimens.

**Fig. 2:**
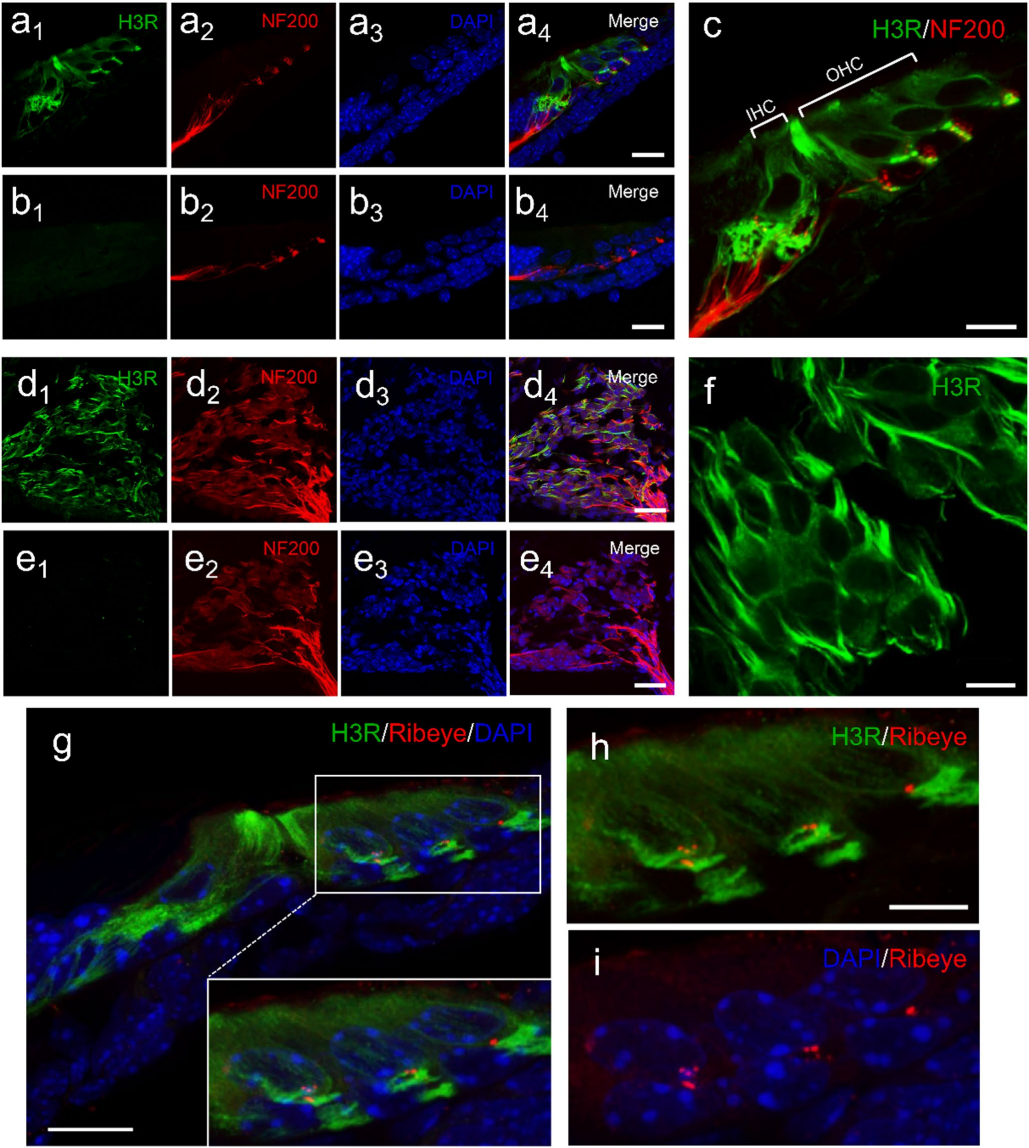
Localization of H3R in hair cells (HCs) and spiral ganglion neurons (SGNs), and its absence at ribbon synapses. **a** Immunofluorescence staining showing H3R expression in both inner and outer HCs within the organ of Corti. **b** Negative control incubated only with the secondary antibody (Alexa Fluor™ 488) without the primary anti-H3R antibody. **c** High-magnification image illustrating H3R signals at the neurite terminals of SGNs near the HCs. **d**–**e** Representative images demonstrating H3R expression in SGNs and the corresponding negative control. **f** High-magnification image showing H3R localization in both the somata and neurites of SGNs. **g**–**i** No colocalization is observed between Ribeye and H3R, indicating the absence of H3R at ribbon synapses. H3R (green); neurofilament 200 (NF200, red in a–f); Ribeye (red in g–i); nuclei (DAPI, blue). Scale bars: 15 μm in **a**–**c**; 20 μm in **d**,** e**,** g**; 5 μm in **f**; 10 μm in **h**,** i**

In the modiolus, H3R was detected in SGN somata and their extending neurites (Fig. 2f), further supporting the RT-qPCR results and highlighting the preferential expression of H3R in cochlear neurons. High-magnification imaging (Fig. 1e) further revealed H3R-positive SGN terminals beneath the outer HCs, specifically at the interface between HCs and adjacent afferent fibers. However, co-immunostaining with Ribeye, a marker for ribbon synapses, indicated no colocalization with H3R (Fig. 2g-i), suggesting that H3R is not directly associated with the presynaptic active zones of cochlear ribbon synapses.

### Ciproxifan Induces Dose- and Region-Dependent Hair Cell Damage in Cochlear Explants

**Fig. 3:**
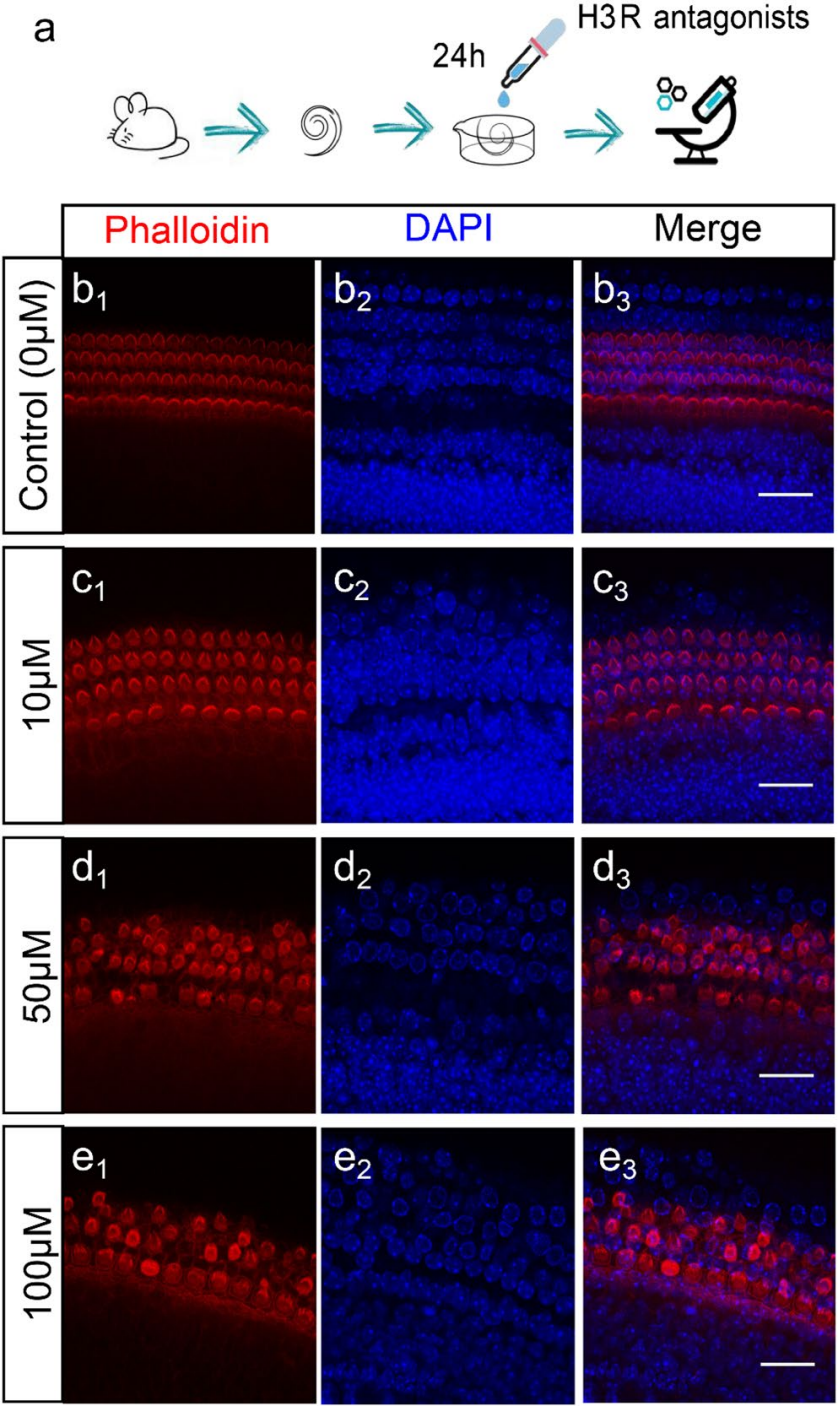
Effects of H3R antagonist ciproxifan on cochlear hair cell (HC) morphology. **a** Schematic illustration of cochlear explant treatment with the H3R antagonists. **b** Representative image of the control group (0µM ciproxifan). **c**–**e** Cochlear explants were treated with ciproxifan at concentrations of 10µM, 50µM, and 100µM, respectively. Representative images shown are from the middle turn of the cochlea. HCs are labeled with phalloidin (red), and nuclei with DAPI (blue). Scale bars: 25 μm

To investigate the functional implications of H3R in the neonatal cochlea, we employed two distinct H3R antagonists: ciproxifan and pitolisant. Cochlear explants, including the organ of Corti and SGNs, were treated with each H3R antagonist at varying concentrations (10µM, 50µM, and 100µM) (Fig. 3a). Following treatment, explants were stained with Alexa Fluor-594 phalloidin to visualize the actin cytoskeleton and stereocilia of HCs. In the control group (0µM), three orderly rows of outer hair cells (OHCs) and a single row of inner hair cells (IHCs) displayed well-preserved stereocilia bundles. DAPI staining confirmed intact nuclear morphology (Fig. 3b). At 10µM ciproxifan, overall HC architecture remained relatively intact. However, minor alterations such as widening and bifurcation of OHC stereocilia were observed (Fig. 3c). At 50µM, HC damage became more pronounced. The OHC rows exhibited disrupted bundle organization, punctate actin condensation, and nuclear shrinkage or loss, indicative of cell loss. Many OHCs displayed collapsed or fragmented stereocilia, with DAPI staining revealing condensed or absent nuclei (Fig. 3d). Treatment with 100 µM ciproxifan resulted in severe HC damage: the typical three-row arrangement of OHCs was disrupted, stereocilia bundles were highly distorted or absent, and in some cases, only cytoskeletal remnants were detected. IHC stereocilia showed marked disorientation and misalignment, with several cells exhibiting dense phalloidin aggregations that obscured the stereocilia structure. Nuclear labeling with DAPI was either markedly condensed or undetectable in these regions (Fig. 3e).

**Fig. 4:**
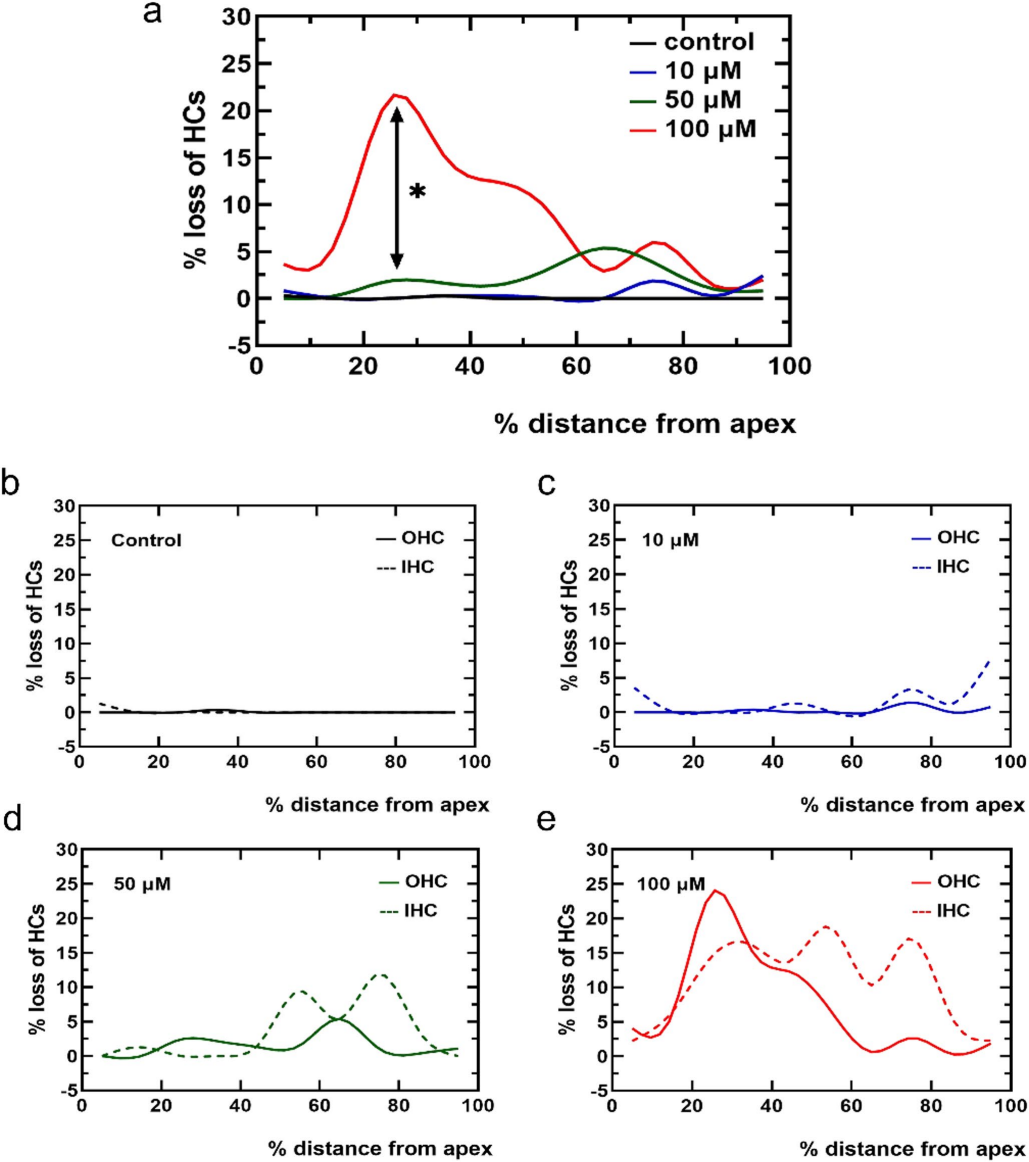
Cochleograms showing hair cell (HC) loss following treatment with varying concentrations of ciproxifan. **a** The cochleogram illustrates the percentage of total missing HCs plotted against the relative distance from the cochlear apex across different ciproxifan concentrations. **b**–**e** Mean cochleograms depicting the percentage of missing inner hair cells (IHCs; dashed lines) and outer hair cells (OHCs; solid lines) along the cochlear length. **b** Control group. **c**–**e** Explants were treated with 10µM (blue), 50µM (green), and 100µM (red) ciproxifan, respectively. Data are presented as fitted curves, with *n* = 6 for each group

To quantitatively assess HC loss along the cochlear axis, cochleograms were generated to evaluate the regional impact of ciproxifan at various concentrations (Fig. 4a). The mean percentage of HC loss was plotted as a function of cochlear length (expressed as percent distance from the apex). A clear dose-dependent pattern of damage was observed. At lower concentrations (10µM and 50µM), HC loss was primarily localized to the basal region (60–80% from the apex). At 100µM, a distinct shift in peak damage was observed toward the apical region (20–30% from the apex), where HC loss reached 21.33%, nearly fivefold higher than the corresponding region in the 50µM group (5.37%) (*p* < 0.05). The control group exhibited negligible HC loss along the entire cochlear length. To further distinguish the vulnerability of IHCs and OHCs, separate cochleograms were generated. At 10µM, a trend toward slightly greater IHC loss compared to OHC loss was observed in the mid-to-basal regions (40–100% from the apex) (Fig. 4c). At 50µM, IHC loss appeared more pronounced in the basal turn, whereas a modest increase in OHC loss was observed in the apical region (20–40%) (Fig. 4d). At 100 µM, OHC damage in the apical turn became the most prominent feature, forming a sharp peak, while IHC loss appeared to predominate beyond 40% from the apex (Fig. 4e). Collectively, these observations suggest that ciproxifan induces both dose-dependent and region-specific damage on cochlear explant HCs.

### The H3R Antagonist Pitolisant Does not Induce Hair Cell Loss in Cochlear Explants

**Fig. 5:**
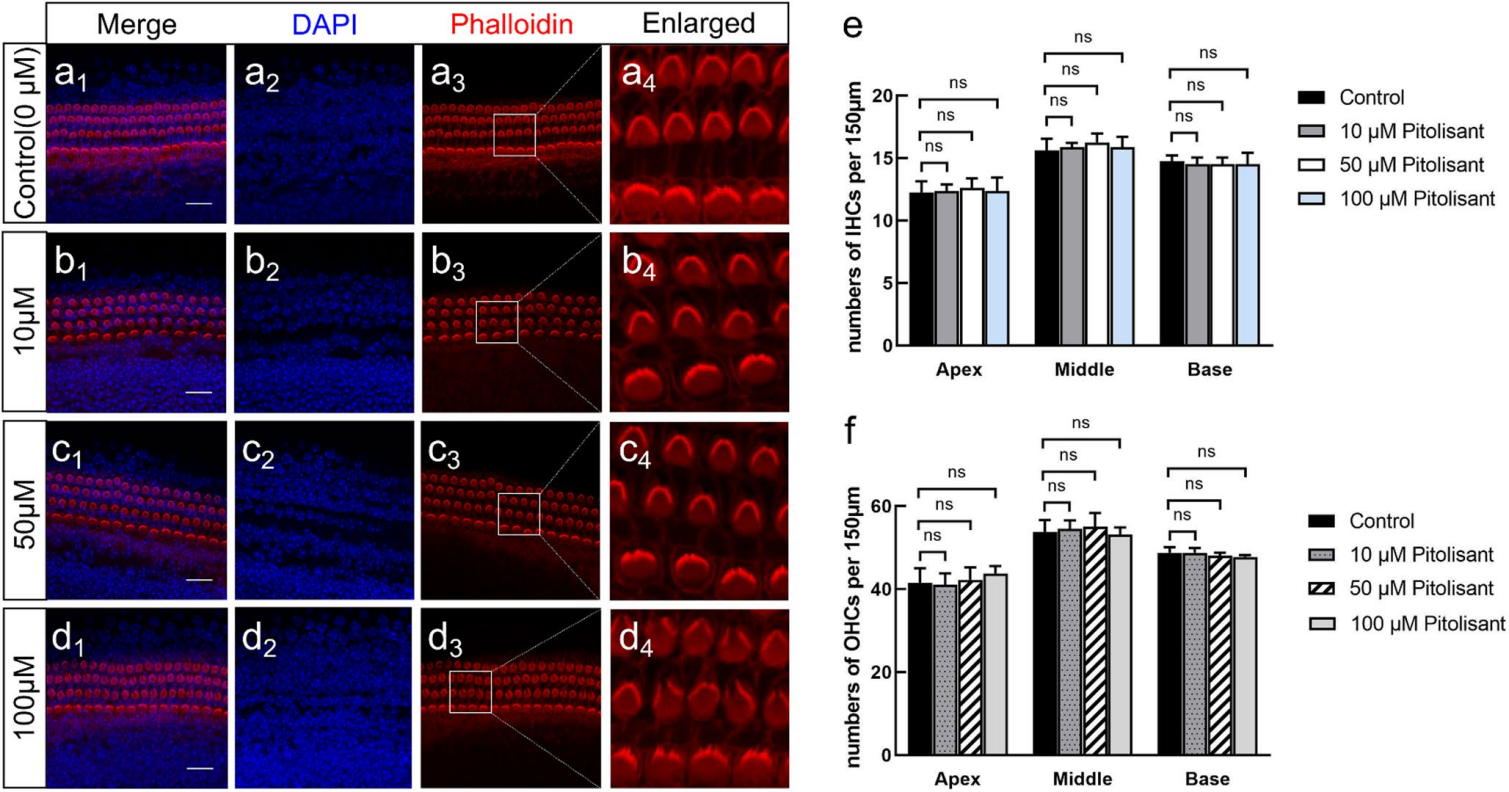
Effects of H3R antagonist pitolisant on cochlear HCs. **a** Representative image of the control group (0µM pitolisant). **b**–**d** Cochlear explants treated with pitolisant at 10µM, 50µM, and 100µM, respectively. **a**_**4**_, **b**_**4**_, **c**_**4**_, **d**_**4**_ High-magnification views of the boxed regions in A_3_-D_3_, respectively. **e**–**f** Quantification of IHCs and OHCs in the apical, middle, and basal turns showed no statistically significant differences between pitolisant-treated groups and controls (*n* = 6 per group). Phalloidin (red), nuclei (DAPI, blue). Scale bars: 25 μm

To determine whether the effects observed with ciproxifan reflect its pharmacological properties or a general H3R-related mechanism, we examined the effects of pitolisant—another well-characterized and highly selective H3R antagonist—under identical experimental conditions. As shown in Fig. 5b–d, cochlear explants treated with 10µM and 50µM pitolisant had HCs with well-preserved architecture, with orderly OHC and IHC rows, intact stereociliary bundles, and no signs of cytoskeletal disorganization or nuclear condensation. However, at a higher concentration of 100µM, structural alterations were noted at the apex of OHC stereocilia bundles, including loss of pointed convergence and bifurcation, resulting in a widened “V” morphology (Fig. 5d_**4**_). These findings suggest pitolisant does not induce HC loss; however, high concentrations may partially affect OHC stereocilia integrity.

Consistent with the morphological findings, quantitative analysis confirmed that pitolisant treatment did not lead to significant HC loss. As shown in Fig. 5e-f, the numbers of IHCs and OHCs in the apical, middle, and basal cochlear turns remained comparable across all pitolisant-treated groups (10µM, 50µM, and 100µM) and controls (*p* > 0.05).

### Biosafety Evaluation of Dimethyl Sulfoxide in Cochlear Explants

**Fig. 6:**
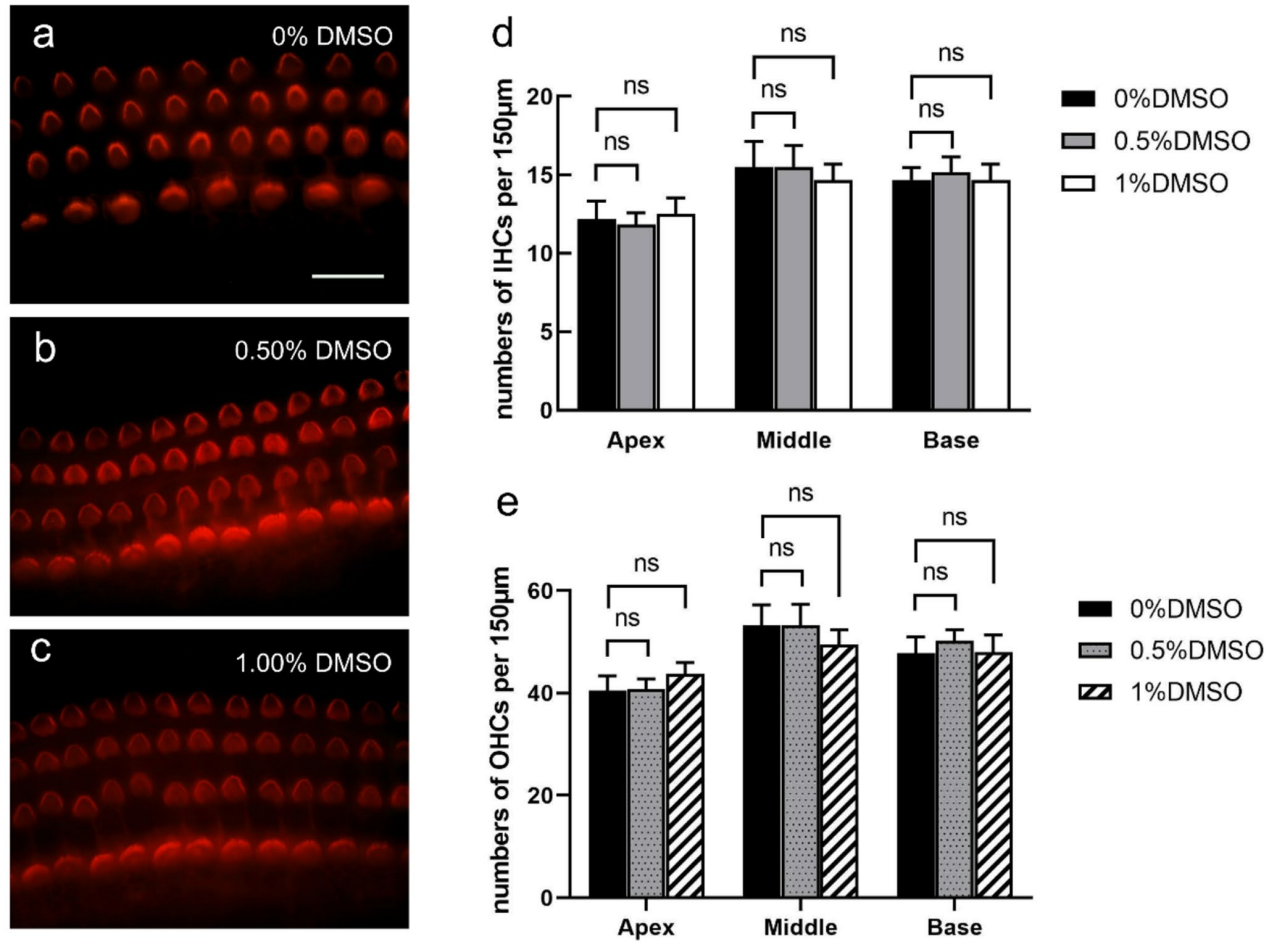
Dimethyl sulfoxide (DMSO) at experimental concentrations does not affect HC viability or morphology. **a** Representative image of the control group cultured in culture medium without DMSO, showing intact HC morphology. **b**–**c** Explants treated with 0.50% and 1.00% DMSO, respectively, exhibited no detectable HC loss or structural alterations. **d**–**e** Quantification of IHCs and OHCs in the apical, middle, and basal turns showed no statistically significant differences between DMSO-treated and control groups. Phalloidin (red), nuclei (DAPI, blue). ns > 0.05; *n* = 6 per group. Scale bar: 50 μm

DMSO is commonly used as a solvent in inner ear research. In this study, DMSO was used to dissolve H3R antagonists [39, 40, 41, 42]. To rule out potential solvent-induced effects and ensure the validity of our experimental results, we evaluated the cytocompatibility of DMSO in neonatal cochlear explants. Explants were incubated under the same conditions in medium containing 0.50% or 1.00% DMSO, both exceeding the concentrations used in the antagonist-treated groups. Immunofluorescence staining showed no morphological abnormalities in HCs. The characteristic arrangement of three rows of OHCs and one row of IHCs was maintained without evidence of cell loss or damage (Fig. 6a-c). Quantitative analysis of IHCs and OHCs in the apical, middle, and basal turns revealed no statistically significant differences compared to untreated controls (0% DMSO) (*p* > 0.05; Fig. 6d-e). These results indicate that DMSO used in this study at concentrations up to 1.00% is well tolerated in cochlear explants and exerts no detectable adverse effects or only negligible effects on cochlear explants.

### H3R Antagonists Induce Aberrant Guidance of Type II Spiral Ganglion Neuron Neurite

**Fig. 7:**
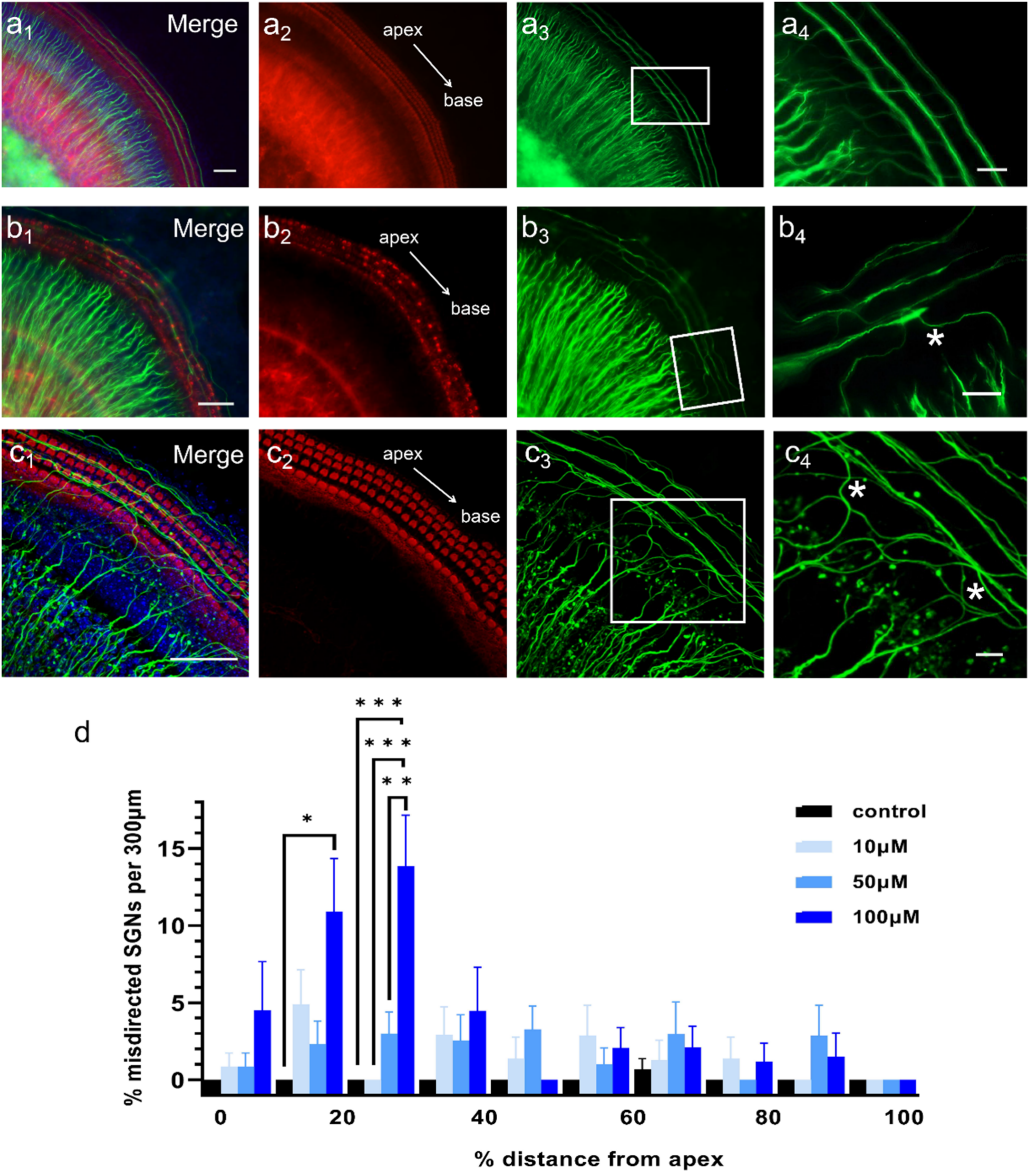
H3R antagonists induce misdirected neurite outgrowth in type II SGNs in cochlear explants. **a** Representative immunofluorescence images showing typical SGN innervation: type I SGNs project radially to IHCs, while type II SGNs extend longitudinally from the apex toward the base to innervate OHCs. **b** Cochlear explant treated with 50µM ciproxifan showing misdirected type II SGN neurites. **c** Cochlear explant treated with 50µM pitolisant showing a similar reversal of type II SGN projections. **a**_**4**_, **b**_**4**_, **c**_**4**_ High-magnification views of the boxed regions in **a**_**3**_, **b**_**3**_, **c**_**3**_. White asterisks indicate misdirected neurites projecting toward the cochlear apex. **d** Percentage of misdirected type II SGNs following treatment with different concentrations of ciproxifan per 300 μm. Data are presented as mean ± SEM. Statistical analysis was performed using one-way ANOVA followed by Bonferroni’s test; **p < 0.01. NF200 (green), phalloidin (red), DAPI (blue); *n* = 6 per group (*n* = 24 in total). Scale bars: 50 μm in **a**_**1–3**_, **b**_**1–3**_, and **c**_**1–3**_; 20 μm in **a**_**4**_, **b**_**4**_, and **c**_**4**_

SGNs are broadly categorized into type I and type II subtypes [43, 44]. Type I SGNs directly innervate IHCs, forming synaptic connections essential for auditory signal transmission [45, 46]. In contrast, type II SGNs extend peripheral neurites toward OHCs, typically following a basal trajectory from the cochlear apex during normal development, guided by specific molecular cues [45]. To investigate whether H3R antagonism affects SGN neurite pathfinding, we examined cochlear explants treated with ciproxifan and pitolisant. In untreated controls, type II SGN neurites followed the expected basal projection pattern (Fig. 7a). In contrast, explants treated with ciproxifan showed occasional misdirected type II neurites projecting toward the cochlear apex (Fig. 7b), deviating from their typical basal orientation, indicating subtle disturbances in neurite guidance. This misdirected growth was consistently observed across all concentrations of ciproxifan (Fig. 7d **and e**). Similarly, treatment with pitolisant also resulted in misdirected type II SGN neurites (Fig. 7c). These findings suggest that H3R signaling regulates the spatial guidance of type II SGN projections during early cochlear development.

We also observed ‘neuritic beading’ of the SGN after 24 h of treatment with 50 µM pitolisant but not ciproxifan (Fig. 7c). This staining pattern indicates damage to the neuronal structure of the SGN. It has previously been associated with neuronal degeneration induced by aging [47], electric field-induced injury [48], and exposure to lead [49].

## Discussion

The present study evaluated the presence and possible function of H3R in the developing cochlea, mapping its distribution and implicating its role in the maturation of the auditory periphery. Extending the work of Takumida et al., we further subdivided the cochlea into three functionally defined regions to quantify *H3R* transcripts [36]. RT-qPCR showed that the amount of *H3R* transcripts is strongly enriched in the modiolus compared with the organ of Corti and lateral wall. The immunofluorescence revealed a robust presence of H3R proteinin SGNs and HCs. These findings corroborate the H3R signal reported in adult CBA/J mice. Still, our high-resolution confocal images did not detect H3R in supporting cells, suggesting either a developmental down-regulation of the receptor in these cells or a strain-specific difference [36]. Interestingly, we detected an H3R signal in the region where the pillar cells are located. However, unlike the consistent staining observed across experiments in the SGNs and HCs, the signal in the pillar cell region was not always apparent, suggesting a possible staining artifact or other technical issues that need to be addressed. Higher magnification and resolution microscopy (e.g., atomic force microscopy or TEM) should be employed to clarify the findings issue.

As the modiolus is a nerve-dense bony core that encases SGN somata and initial axon segments—the gateway for encoding and relaying acoustic information [45]—the high H3R abundance in this region points to a potential role in SGN maturation and the regulation of neuronal excitability. Previous studies have suggested that histamine may regulate cell proliferation, differentiation, and synaptic plasticity across various systems [50, 51, 52]. The H3R receptor is predominantly located in the central nervous system, playing a key role in neurotransmitter release and the regulation of synaptic plasticity [50, 51]. Given the abundance of histamine in mast-cell granules and the identification of mast cells in rodent cochleae [31, 32, 34], along with the recent detection of cochlear HDC [35], our data strengthen the view of an endogenous histamine–H3R axis participating in cochlear homeostasis during the critical postnatal period before hearing onset.

Our comparison of the two H3R antagonists revealed strikingly divergent outcomes. Ciproxifan induced dose-dependent and region-specific HC loss, whereas pitolisant, even at 100 µM, elicited only stereociliary distortion without HC loss. Since both drugs are high-affinity H3R antagonists/inverse agonists [52, 53, 54], these differences are unlikely to result from simple H3R blockade; instead, they suggest compound-specific pharmacological actions, such as differing drug affinities, with ciproxifan having a higher affinity for the mouse H3R (Ki 0.5–0.8 nM) [55] than pitolisant (Ki 1.3 µM) [56]. Moreover, pitolisant, in addition to H3R, also binds to the sigma-1 receptor, which modulates endoplasmic reticulum stress and calcium signaling [57]. Sigma-1 receptor is expressed by cochlear HCs and SGNs, and upon activation, it provides neuro- and otoprotective effects [58]. Thus, the impact of the pitolisant-sigma-1-induced pathway could potentially counteract the H3R-induced ototoxic pathway. Furthermore, in contrast to ciproxifan, pitolisant also binds to the sigma-2 receptor (present in SGN), the serotonin receptor 5HT2A, and the dopamine receptor D3. However, studies have not confirmed the expression of D3 [59] nor addressed the expression of sigma-2 or 5HT2A in the cochlea of mice.

Cochleograms revealed that ciproxifan-induced HC damage follows a region-specific pattern. This pattern might be related to the varying expression levels of H3R in different cochlear regions. Several biophysical features of HCs change progressively from base to apex—bundle height, resting MET conductance, Ca²⁺-buffering capacity, and synaptic ribbon size—factors that set frequency tuning and influence stress vulnerability [60, 61]. SGNs also exhibit tonotopic gradients in synaptic protein composition [62]. If H3R expression follows a similar gradient, apical HCs may encounter increased antagonist occupancy and higher off-target stress, which explains their increased susceptibility. These findings underscore the need to consider compound-specific pharmacology and cochlear regional biology when targeting H3R pathways for therapeutic ends.

This study’s further key observation was the rapid deformation of HC stereocilia after H3R antagonism. Within 24 h, both antagonists induced rapid V-bundle bifurcation and misalignment, indicating a loss of actin-core integrity. Although stereociliary F-actin is remarkably stable, local repair is initiated once damage occurs, and successful maintenance relies on a finely balanced cycle of filament severing, polymerization, and re-bundling [63, 64]. Actin-depolymerizing factor (ADF) and cofilin-1 (CFL1) are central to this process: they sever and recycle older filaments, providing barbed-end substrate for espin-mediated re-bundling [65]. We therefore propose that H3R signaling helps to maintain bundle architecture by sustaining the activity of ADF/CFL1. Acute pharmacological antagonism could affect this balance, shifting the local actin economy towards net disassembly and producing the bundle bifurcation we observed. Interestingly, the splaying and partial collapse of stereocilia bundles in our samples resemble features described in radixin-deficient mice, where cochlear stereocilia undergo progressive postnatal degeneration due to impaired membrane–cytoskeleton anchoring [66]. Ezrin–Radixin–Moesin (ERM) proteins play a crucial role in maintaining the structure of stereocilia by linking the actin core to the apical membrane [67]. Their loss disrupts bundle cohesion and impairs the polarized localization of membrane proteins [68]. Similarly, reduced phosphorylation of ERM proteins, including ezrin, has been associated with stereocilia disorganization and decreased synapse density during postnatal HC development in Pak1 knockout mice [69]. Although our model involves pharmacological intervention rather than genetic manipulation, it suggests that H3R signaling could influence ERM function or localization. Since ERM-mediated anchoring is highly sensitive to intracellular signals, temporary disruption might explain the observed morphology. Further research is needed to determine whether H3R antagonism affects ERM proteins or other cytoskeletal regulators in cochlear HCs.

Besides highlighting the potential role of H3R in maintaining HC stereocilia, our study indicates a possible function for H3R in inner ear neural development. The abnormal type II SGN neurite projections seen in cochlear explants treated with H3R antagonists suggest that H3R may be essential for the proper spatial projection of SGN axons. Neurite targeting in the neonatal cochlea is sculpted by a combinatorial code of neurotrophins, cell-adhesion molecules, and ephrin–Eph gradients [70, 71, 72]. Histamine signaling could intersect with this code in several ways. One possibility is that mast-cell histamine—already documented in the cochlea [31]—enhances brain-derived neurotrophic factor (BDNF) release [73], such that H3R on mast cells or SGN terminals modulates the local BDNF/NT-3 milieu that regulates axon growth. Additionally, H3R functions as a presynaptic GPCR that adjusts transmitter release and intracellular cAMP [74, 75]; by analogy with vestibular circuits [76], H3R could fine-tune glutamatergic signaling between nascent HC–SGN synapses, providing activity-dependent cues for branch stabilization or elimination. Whether these effects are mast-cell driven, neuron-intrinsic, or both will require targeted genetic or pharmacological tests.

The clinical applications of histamine receptor modulators have been extensively explored, with H3R-targeting agents showing particular promise in the treatment of neurological disorders. Pitolisant—the first H3R approved for human use (Wakix^®^)—is indicated for adults and children 6 years and older with narcolepsy [77, 78]. Our findings, however, urge caution when extending such therapy to specific populations, such as pregnant women. Because histamine signaling is integral to early inner-ear maturation, systemic H3R blockade during pregnancy could theoretically disturb immune-neural crosstalk in the fetal cochlea and produce lasting auditory deficits. Although the micromolar antagonist concentrations used in our explants exceed typical therapeutic plasma levels, they reveal a plausible hazard pathway. Until dedicated teratology and pediatric safety studies are completed, clinicians should weigh the proven central nervous system benefits of H3R inverse agonists against the still uncertain risk of developmental ototoxicity, particularly in pregnant patients or inoff-label pediatric applications.

Our study is not free of pitfalls. First, focusing on P4–P5 explants captures only a brief developmental window; examining embryonic stages and post-hearing ages will reveal whether H3R functions change over time. Second, our micro-dissection partially shears the modiolus and may under-sample some SGN subtypes. Using whole-cochlea organotypic cultures or in vivo models should give a more complete picture of neuronal phenotypes. Third, while our data suggest ciproxifan-driven oxidative and epigenetic stress, we have not yet directly demonstrated these mechanisms. Integrating transcriptomics with targeted redox assays and chromatin analyses will be essential to define how these pathways converge on HC vulnerability. Another limitation of our study is the use of high concentrations of both H3R antagonists. Published data indicate that in mice receiving 1 mg/kg of ciprofixan either intraperitoneally or orally, the peak serum concentration was 1.425 µM and 0.890 µM, respectively. In cats, the serum levels of ciproxifan after an oral dose of 3 mg/kg were 5.07 µM [79]. In humans, indirect measurements indicated a nanomolar range of ciproxifan serum concentration [80]. Similarly, the serum concentration of pitolisant was 0.0115 µM in mice and 0.067 µM in humans [81]. This indicates that the concentrations we used in our experiments were within and above that range, aligning with current toxicology protocols [82]. Finally, using established auditory cell markers such as myosin VII for hair cells [83], peripherin for type II SGN [84], or CD44 for pillar cells [85] in combination with high-resolution microscopy should help answer the still-open questions.

## Conclusions

This study investigated the expression and function of H3R in the cochleae of P4-5 C57BL/6 mice, highlighting the following key findings:

- H3R is expressed in SGNs and HCs, with significantly higher mRNA expression levels in the modiolus compared to the organ of Corti and the lateral wall.
- Ciproxifan caused dose- and region-dependent HC loss.
- Both tested H3R antagonists caused structural abnormalities in HC stereocilia, suggesting a crucial role for H3R signaling in maintaining stereocilia integrity.
- Antagonist exposure redirects type II SGN neurites toward the apex, implicating H3R in cochlear axon guidance.
- DMSO concentrations (0.025% and 0.050%) utilized as a solvent exhibited no cytotoxicity toward HCs, confirming its suitability and biological safety under experimental conditions.

Our findings identify H3R as a new lever for controlling stereocilia maintenance and SGN pathfinding in the neonatal cochlea. Genetic knockout models and high-resolution in vivo imaging will be essential next steps in mapping the downstream pathways and evaluating H3R-targeted therapies for hearing disorders.

## Data Availability

The data supporting this study’s findings are available from the corresponding author upon reasonable request.
